# The influence of the shape of Au nanoparticles on the catalytic current of fructose dehydrogenase

**DOI:** 10.1007/s00216-019-01944-6

**Published:** 2019-07-08

**Authors:** Paolo Bollella, Yuya Hibino, Paolo Conejo-Valverde, Jackeline Soto-Cruz, Julián Bergueiro, Marcelo Calderón, Oscar Rojas-Carrillo, Kenji Kano, Lo Gorton

**Affiliations:** 1 Department of Chemistry and Biomolecular Science, Clarkson University, Potsdam, NY 13699 USA; 2 Division of Applied Life Sciences, Graduate School of Agriculture, Kyoto University, Sakyo, P.O. Box 86, Kyoto, 606-8502 Japan; 3 Chemistry School, Universidad Nacional, P.O. Box 86-3000, Heredia, Costa Rica; 4 Institut für Chemie und Biochemie, Freie Universität Berlin, Takustrasse 3, 14195 Berlin, Germany; 5 POLYMAT and Applied Chemistry Department, Faculty of Chemistry, University of the Basque Country UPV/EHU, Paseo Manuel de Lardizabal 3, 20018 Donostia-San Sebastián, Spain; 6 Department of Analytical Chemistry/Biochemistry, Lund University, P.O. Box 124,, Lund, 221 00 Sweden

**Keywords:** Fructose dehydrogenase (FDH), Gold nanotriangles (AuNTrs), Gold nanoparticles (AuNPs), Nanoparticle shape, Direct electron transfer (DET)

## Abstract

Graphite electrodes were modified with triangular (AuNTrs) or spherical (AuNPs) nanoparticles and further modified with fructose dehydrogenase (FDH). The present study reports the effect of the shape of these nanoparticles (NPs) on the catalytic current of immobilized FDH pointing out the different contributions on the mass transfer–limited and kinetically limited currents. The influence of the shape of the NPs on the mass transfer–limited and the kinetically limited current has been proved by using two different methods: a rotating disk electrode (RDE) and an electrode mounted in a wall jet flow-through electrochemical cell attached to a flow system. The advantages of using the wall jet flow system compared with the RDE system for kinetic investigations are as follows: no need to account for substrate consumption, especially in the case of desorption of enzyme, and studies of product-inhibited enzymes. The comparison reveals that virtually identical results can be obtained using either of the two techniques. The heterogeneous electron transfer (ET) rate constants (*k*_S_) were found to be 3.8 ± 0.3 s^−1^ and 0.9 ± 0.1 s^−1^, for triangular and spherical NPs, respectively. The improvement observed for the electrode modified with AuNTrs suggests a more effective enzyme-NP interaction, which can allocate a higher number of enzyme molecules on the electrode surface.

**Electronic supplementary material:**

The online version of this article (10.1007/s00216-019-01944-6) contains supplementary material, which is available to authorized users.

**Graphical abstract:**
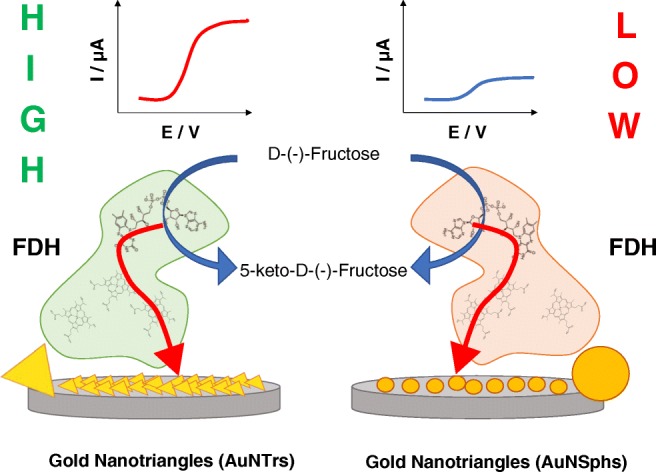
The shape of gold nanoparticles has a crucial effect on the catalytic current related to the oxidation of D-(-)-fructose to 5-keto-D-(-)-fructose occurring at the FDH-modified electrode surface. In particular, AuNTrs have a higher effect compared with the spherical one.

## Introduction

Nanostructuration of electrodes seems to play a crucial role in the development of biodevices, such as biosensors and enzymatic fuel cells (EFCs) [1, 2], which are based on a direct electron transfer (DET) communication between the biological material and the electrode [3, 4]. In most cases, redox enzymes immobilized onto “planar” electrodes show slow electron transfer (ET) rate constants between the redox cofactor and the unmodified electrode and very small electrocatalytical currents in the presence of substrate, while gold nanoparticles (AuNPs) offer the possibility to wire the redox protein to the electrode producing a favorable orientation improving so far both the ET rate and the electrocatalytical current [5, 6].

Many papers present effective bioelectrocatalytical processes, where several mono- and multi-cofactor redox enzymes [7], such as cellobiose dehydrogenase [5, 8, 9], horseradish peroxidase, superoxide dismutase, fructose dehydrogenase [10–12], blue multicopper oxidases (MCOs) [13, 14], and human sulfite oxidase [15], have been immobilized onto nanostructured electrodes. Despite the large interest in AuNPs, there are only few reports about the influence of the size (e.g., diameter) and shape of the NPs on the enzymatic reactions occurring at the electrodes, sometimes improving the ET rate or changing the mechanism of the bioelectrocatalytic process [16, 17].

Fructose dehydrogenase (FDH, EC 1.1.99.11) from *Gluconobacter japonicus* has been widely studied to develop biosensors based on mediated electron transfer and DET as well as bioanodes for enzymatic fuel cells (EFCs) [18, 19]. FDH from *Gluconobacter japonicus* NCBR 3260 is a membrane-bound flavocytochrome oxidoreductase also belonging to the hemoflavoprotein family and is a heterotrimeric membrane-bound enzyme complex with a molecular mass of 146.4 kDa, consisting of three subunits, viz. subunit I (DH_FDH_), which is the catalytic dehydrogenase domain with a covalently bound flavin adenine dinucleotide (FAD) cofactor, where d-(-)-fructose is involved in a 2H^+^/2e^−^ oxidation to 5-dehydro-d-(-)-fructose; subunit II (CYT_FDH_), a cytochrome domain acting as a built-in electron acceptor with three heme *c* moieties covalently bound to the enzyme scaffold and two of them are involved, one by one, in the electron transfer pathway; and subunit III, which is not involved in the electron transfer but plays a key role for the stability of the enzyme complex [20, 21].

The suggested electron transfer pathway for FDH when it is immobilized on the electrode surface and in the absence of any competing e^−^ acceptors [22] goes initially through the oxidation of d-(-)-fructose to form 5-keto-d-(-)-fructose and involves a net 2e^−^/2H^+^ transfer with the reduction of FAD to form FADH_2_. It then further proceeds with a partial reoxidation of FADH_2_ to FADH·, through a first internal electron transfer (IET) through two of the three heme *c*:s contained in subunit II in direct contact with the electrode surface at which these heme *c*:s are reoxidized. Finally, the reoxidation of FADH· to FAD gives the second internal electron transfer (IET) reaction through the two involved heme *c*:s, which in turn are reoxidized at the electrode surface [18]. Recently, several researchers managed to demonstrate that the third heme *c* is not involved in the ET process, due to its distance from the other two heme *c*:s contained in subunit II, making the latter step of the ET process energetically unfavored [23–25]. Therefore, the electrons are directly transferred from the second heme *c* to the electrode [23, 26].

The DET reaction between FDH and electrodes has been demonstrated in a large number of publications, immobilizing the enzyme on different electrode materials including both polycrystalline gold electrodes [27] as well as on nanomaterials like single- or multi-walled carbon nanotubes [28] and other carbon nanostructures [29–32] and gold nanoparticles [33] or by exploiting several immobilization approaches such as self-assembling monolayers (SAMs) [34, 35], polymers, and other cross-linking agents [27]. Moreover, also mediated electron transfer (MET) reactions for FDH were exploited to develop various amperometric biosensors [36–48].

In the last decades, many researchers devoted considerable attention toward the synthesis and the application of AuNPs in several fields of chemistry [49]. Among all kinds of nanomaterials, AuNPs play an important role in making electrode modifications, because of their high surface area-to-volume ratios and high surface energy, which facilitate the immobilization of several kinds of proteins, allowing to act as electron conducting pathways between the prosthetic groups of the enzymes and the electrode surface [49–51]. Several methods for synthesis of AuNPs have been reported in the literature considering different reducing agents like citric acid, NaBH_4_, surfactants, reducing sugars, and polyphenols [52–56]. In our previous paper, we reported on the synthesis of metal nanoparticles (MNPs) using quercetin as reducing and stabilizing agent at room temperature [57]. Besides the classical methods and green chemistry–based methods, also surfactants like dimyristoyl-l-phosphatidyl-dl-glycerol (DMPG), alkyltrimethylammonium bromides, or cetylpyridinium chloride have been widely employed [58, 59]. Usually, MNPs can have several 2D or 3D shapes like triangles, spheres, hexagons, and cubes exhibiting different chemical and physical properties in heterogeneous catalysis [60].

In this paper, graphite electrodes have been modified with triangular (AuNTrs) and spherical gold (AuNSphs) nanoparticles to investigate whether the shape of the NPs can affect both the mass transfer–limited and the kinetically limited currents by using two different methods: a rotating disk electrode (RDE) and an electrode mounted in a wall jet flow-through electrochemical cell attached to a flow system. The differently prepared electrodes were further modified by drop-casting FDH directly on the top of the AuNTrs/G and AuNSphs/G electrodes. Finally, both methods allowed quantifying the influence of the shape of the NPs on the mass transfer–limited current and the heterogeneous electron transfer rate constant (*k*_S_).

## Experimental

### Chemicals

d-(-)-Fructose, sodium acetate (NaAc), spherical gold nanoparticles (AuNSphs, *d* = 100 nm stabilized in citric acid), HAuCl_4_, hydrochloric acid (HCl), sodium hydroxide (NaOH), H_2_SO_4_, and sodium dodecyl sulfate (SDS) were purchased from Sigma-Aldrich (St. Louis, MO, USA). The phospholipids, 1,2-dimyristoyl-sn-glycero-3-phospho-rac-glycerol sodium salt (DMPG-Na), and phosphatidylcholine (PC) were kindly donated by LIPOID AG (Germany). d-Fructose dehydrogenase from *Gluconobacter japonicus* (FDH; EC 1.1.99.11) was purified from the culture supernatant of *Gluconobacter japonicus* NBRC 3260 obtained from the National Institute of Technology and Evaluation (Nishinomiya, Hyogo Pref., Japan), and solubilized in PBS buffer pH 6 (50~500 mM) containing 0.1 mM 2-mercaptoethanol and 0.1% *v*/*v* Triton X-100 (volumetric activity measured with potassium ferricyanide at pH 4.5 = 420 ± 30 U mL^−1^, specific activity = 250 ± 30 U mg^−1^, protein concentration = 1.7 ± 0.2 mg mL^−1^) [20]. All solutions were prepared using Milli-Q water (*ρ* = 18.2 MΩ cm at 25 °C; total organic compounds (TOC) < 10 μg L^−1^, Millipore, Molsheim, France).

### Buffer exchange for spherical gold nanoparticles

Five milliliters of a solution containing spherical gold nanoparticles (AuNSphs, *d* = 100 nm stabilized in citric acid) was centrifuged by using Zeba™ Spin Desalting Columns, 7K MWCO (Thermo Fisher, Life Technologies Europe BV, Stockholm, Sweden). The AuNSph pellet was washed 5 times with Milli-Q water to eliminate all traces of citric acid and redispersed in a 0.150 M SDS aqueous solution. Finally, the AuNSph suspension was stored at 4 °C.

### Synthesis, purification, and characterization of gold nanotriangles

A mixture of phospholipids, namely DMPG-Na/PC, was dispersed in water with a ratio of 1:1 *w*/*w* and stirred for 72 h at room temperature. Next, the dispersion was sonicated for 1 min. Next, 250 μL of a 2 mM tetrachloroaurate solution was added to the phospholipid mixture and gently stirred overnight in a water bath at 25 °C. The color change from yellow to dark red indicated the formation of AuNPs.

After the reaction was completed, the nanoparticles were centrifuged for 1 h at 4400 rpm. The supernatant was removed and the particles were redispersed in water. The procedure was performed twice centrifuging for 30 min at 4400 rpm. Further, the solution containing a mixture of gold nanotriangles (AuNTrs) and byproducts (spherical particles) was redispersed in 2 mL of pure water and purified by using a depletion-induced flocculation method. The separation of NPs based on shape and size can be obtained by tuning the surfactant micelle concentration to create an entropic, short-ranged depletion attraction between the NPs, resulting in a preferential aggregation and sedimentation of one kind of nanoparticles, leaving the others in solution [61]. Therefore, the depletion-flocculation separation was performed in the presence of SDS micelles at an optimum concentration of 0.150 M of the final solution. The flocculation was completed overnight, removing the supernatant containing the AuNSphs while the precipitated AuNTrs were washed and resuspended in Milli-Q water.

The purified AuNTrs were characterized by dynamic light scattering (DLS). Measurements were carried out at 25 °C at a fixed angle of 173° (“backscattering detection”) by using a Nano Zetasizer (Zetasizer Nano ZS90, Malvern Instruments Ltd, Malvern, UK) equipped with a He–Ne laser (*λ* = 633 nm; 4 mW) and a digital autocorrelator. The zeta potential was determined by means of the Nano Zetasizer based on the electrophoresis principle. Micrographs of NPs formed in the phospholipid dispersion were taken with a Hitachi HT7700 transmission electron microscope (TEM) operated at 100 kV (Hitachi Europe GmbH, Düsseldorf, Germany). Transmission and scanning electron microscopy of purified samples were prepared by blotting the NPs on carbon-coated copper grids and visualized by using a TEM detector on a Hitachi Scanning Microscope (SU8030) at 20–30 kV (Hitachi Europe GmbH, Düsseldorf, Germany). UV-Visible-NIR spectra were recorded with a Shimadzu UV-Vis-NIR-3600 Plus spectrophotometer (Kyoto, Japan) with samples contained in a quartz cuvette, operating at a resolution of 1 nm from 300 to 1100 nm.

### Electrochemical measurements

Graphite rods (Alfa Aesar GmbH & Co KG, AGKSP grade, ultra “F” purity, and 3.05 mm diameter, Karlsruhe, Germany) were polished on wet emery paper (Turfbak Durite, P1200) and then carefully rinsed with Milli-Q water. The enzyme-modified electrodes were modified respectively with 5 μL of AuNTrs or 5 μL of AuNSphs and left to dry at room temperature. After, 5 μL of a d-fructose dehydrogenase solution was drop-cast and allowed to physically adsorb on the top of the modified graphite rod electrodes, overnight at 4 °C. Cyclic voltammetry (CV) experiments were carried out using an Autolab potentiostat (model PGSTAT30, Metrohm Autolab B.V. Ecochemie, Utrecht, The Netherlands) equipped with GPES, version 4.9. A conventional three-electrode electrochemical cell was used for all experiments performed with an Ag|AgCl (sat. KCl) as reference electrode, a platinum wire as counter electrode, and a modified graphite electrode as working electrode. The rotating disk electrode (RDE) experiments were carried out using a 616A Electrode Rotator (EC&CG Princeton, GammaData Instruments AB, Uppsala, Sweden). All the CV experiments were carried out under temperature control by using the thermostated electrochemical cell (Cat. 6.1418.150, Metrohm AB, Bromma, Sweden) and a cryostatic bath (*T* ± 0.01 °C, LAUDA RM6, Delran, NJ, USA). Flow through measurements were performed using an analogue potentiostat (Zäta Elektronik, Höör, Sweden) connected with a strip chart recorder (Kipp & Zonen, Utrecht, The Netherlands). The modified graphite electrode, an Ag|AgCl (0.1 M KCl) reference electrode, and a Pt wire counter electrode were fitted into a wall jet cell. The electrochemical system was equipped with a flow system consisting of a peristaltic pump (Gilson, Villier-le-Bel, France) and a six-port valve electrical injector (Rheodyne, Cotati, CA, USA) [62].

## Results and discussion

### UV-Vis-NIR and TEM characterization of gold nanotriangles

**Fig. 1:**
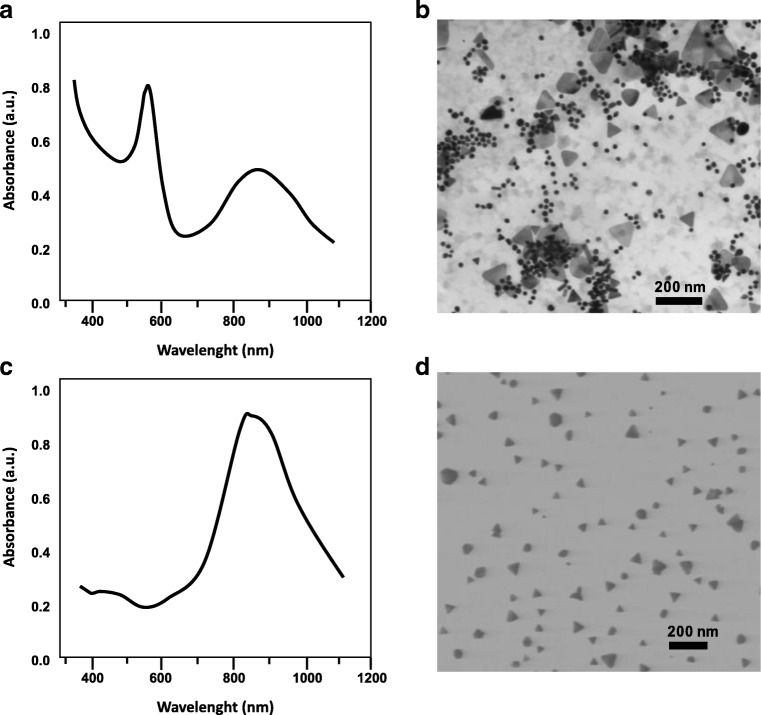
**a** UV-Vis-NIR spectra before the purification. **b** TEM picture of AuNTrs before the purification. **c** UV-Vis-NIR spectra after the purification. **d** TEM picture of AuNTrs after the purification

The synthesis of gold nanotriangle (AuNTr) using a phospholipid mixture as a reducing and stabilizing agent in water is relatively simple and reproducible. The mass ratio between the metal precursor and DMPG-Na/PC was kept constant (1:4) during the synthesis at 25 °C. Under such conditions, the solution turns deep red showing two UV-Vis absorption maxima at 523 nm and 870 nm, as reported in Fig. 1a (before purification), characteristic for the formation of spherical small dimension AuNPs and anisotropic NPs, respectively. The AuNTrs were characterized using transmission electron microscopy before (Fig. 1b) and after purification (Fig. 1d). By considering these TEM pictures, a shape yield higher than 95% was demonstrated, resulting in a green color solution due to the concentration of AuNTrs with sizes of about 50 nm and 200 nm after only one sedimentation and re-dispersion step. Consequently, a maximum at about 870 nm is observed in the UV-Vis-NIR spectrum (Fig. 1c, after purification) confirming that highly concentrated AuNTrs are present. Similar results have been observed by Liebig et al. [63] and Scarabelli et al. [64], using AOT and CTAC micelles, respectively, during the purification of AuNTr obtained by different approaches. As expected, the AuNTrs show a negative zeta potential of − 60 mV as a result of the coating with anionic surfactants (data not shown). Furthermore, AuNSphs were also characterized using transmission electron microscopy (TEM) as reported in Fig. S3 (see Electronic Supplementary Material, ESM), showing a good size distribution.

### Electrochemical characterization of FDH-modified graphite electrode

CV experiments were performed with modified graphite electrodes in absence and in presence of substrate in order to assess the contribution of the electrode nanostructuration using differently shaped AuNPs (spherical and triangular) on the catalytic current related to the oxidation of d-(-)-fructose to 5-keto-d-(-)-fructose catalyzed by FDH.

**Fig. 2:**
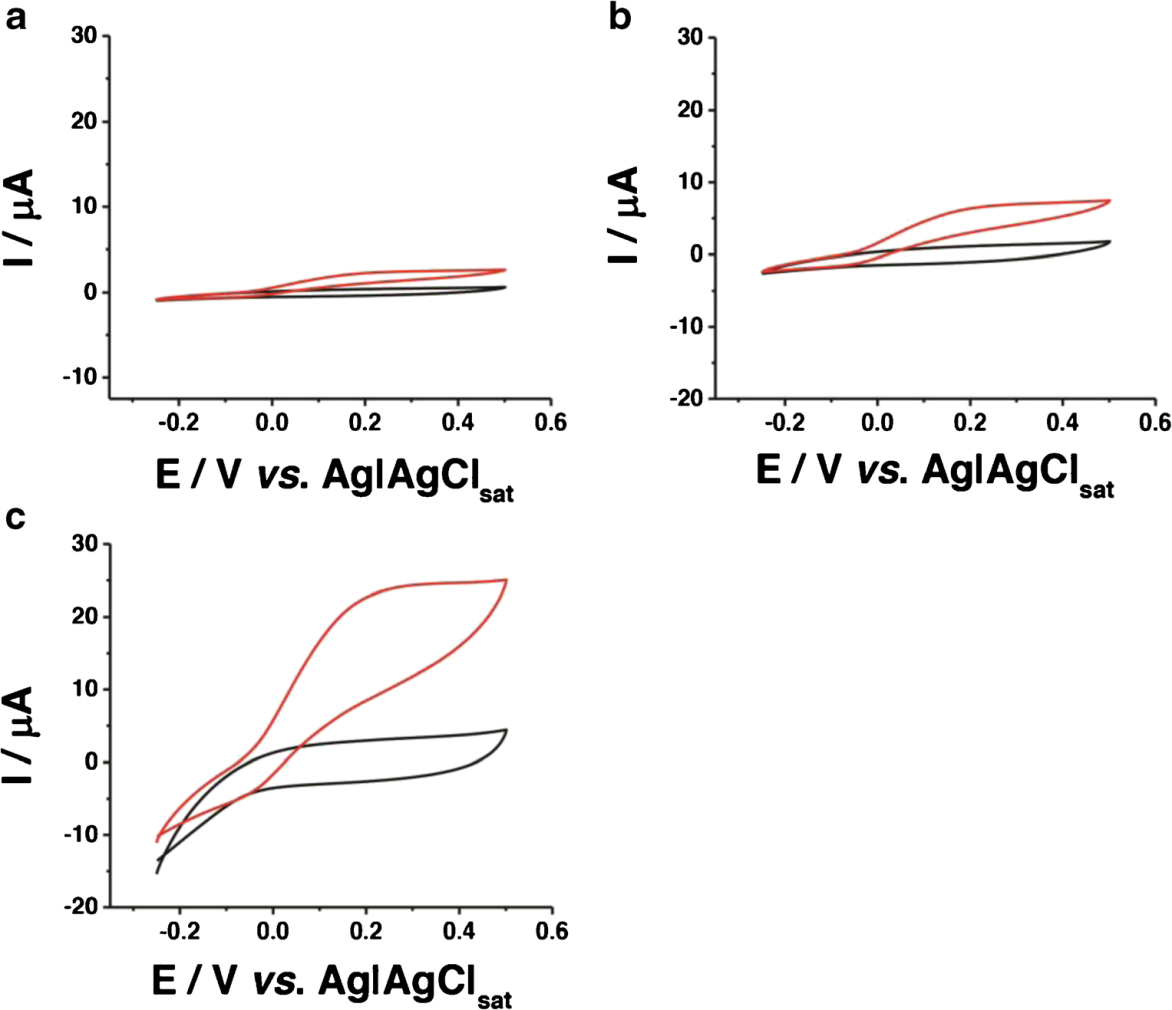
CVs performed in 50 mM NaAc buffer pH 4.5 in absence (black curves) and in presence of 1 mM d-(-)-fructose (red curves) for **a** FDH/G, **b** FDH/AuNSphs/G, and **c** FDH/AuNTrs/G at scan rate 5 mV s^−1^. The measurements were carried out after 20 min N_2_ degassing and *T* = 25 °C

Figure 2a shows the CVs for a FDH/G-modified electrode in 50 mM NaAc buffer pH 4.5 in the absence (black curve) and in the presence (red curve) of 1 mM d-(-)-fructose. From these CVs, it is clear that the non-turnover case reveals no apparent electroactivity of FDH, and in the presence of fructose, there is only a slight electrocatalytic wave with an onset potential, *E*_ONSET_, at − 0.050 V vs. Ag|AgCl_sat_ rising up to maximum current of 2.5 μA at 0.2 V vs. Ag|AgCl_sat_. The low catalytic current is probably due to a combination of the low roughness of the electrode surface and the random orientation of the enzyme onto the electrode surface.

Figure 2b depicts CVs for a FDH/AuNSphs/G-modified electrode in non-turnover conditions (black curve) (50 mM NaAc buffer pH 4.5), showing surprisingly no redox waves related to DET of CYT_FDH_. However, in turnover conditions (1 mM d-(-)-fructose, red curve), the modified electrode showed a higher electrocatalytical wave compared with FDH/G, starting at *E*_ONSET_ = − 0.070 V vs. Ag|AgCl_sat_ rising up to 7.6 μA at 0.2 V vs. Ag|AgCl_sat_. In this case, the increase in the electrocatalytical wave is probably due to the enhanced real surface area of the modified electrode, which allows a higher enzyme loading. Nevertheless, it should be taken into account also the SDS layer onto the nanoparticles, which surprisingly creates a favorable environment for the immobilization of FDH.

Finally, Fig. 2c shows the CVs for the FDH/AuNTrs/G-modified electrode in 50 mM NaAc buffer pH 4.5; however, also here, there are no evident redox waves for the DET reaction of CYT_FDH_. The CV in the presence of substrate (red curve) showed the highest electrocatalytical current compared with the others with an *E*_ONSET_ starting at − 0.103 V vs. Ag|AgCl_sat_ rising up to 22.5 μA at 0.2 V vs. Ag|AgCl_sat_. The result is probably ascribable to the efficient packing of AuNTrs onto the electrode surface, which would sensibly enhance the enzyme loading and also the ET rate constant.

Since both kinds of spherical and triangular NPs were covered with a layer of SDS creating an unexpected favorable immobilization environment, we considered a possible influence of the shape of the NPs on the catalytic current caused by FDH [65]. In particular, Compton and his co-workers published a paper reporting on the diffusion-limited currents to NPs of various shapes supported on an electrode by means of a mathematical simulation on spherical and hemispherical shapes [66]. For this reason, we believe that the different behaviors between the FDH/AuNSphs/G and FDH/AuNTrs/G could be explained by the influence of the shape of the NPs on the limiting kinetic current part of the catalytic current. Therefore, in the section below, we reveal the results of our investigation of the influence of the shape of the NPs on the diffusion-limited current related to the catalytic oxidation of d-(-)-fructose by using two different approaches: the rotating disk electrode (RDE) and flow through amperometric wall jet cell [67–69].

### The dependence of mass transfer–limited current on the shape of the NPs: rotating disk electrode and flow through amperometric wall jet cell studies

The reaction of FDH with fructose starts with the oxidation of d-(-)-fructose to form 5-keto-d-(-)-fructose, which corresponds to the 2e^−^/2H^+^ reduction of FAD to FADH_2_ followed by the first internal electron transfer through two cyt *c* moieties (one heme *c* is not involved at all) contained in subunit II for the partial regeneration of FADH· in its semi-oxidized state and the delivery of the 1st e^−^, as reported below (Eqs. (1–4)):

1 $$ \mathrm{D}-\left(-\right)-\mathrm{fructose}+\mathrm{FAD}\to 5-\mathrm{keto}-\mathrm{D}-\left(-\right)-\mathrm{fructose}+{\mathrm{FADH}}_2 $$

2 $$ {\mathrm{FADH}}_2+\mathrm{cyt}{c}_1-{\mathrm{Fe}}^{3+}\to {\mathrm{FADH}}^{\cdotp }+\mathrm{cyt}{c}_1-{\mathrm{Fe}}^{2+} $$

3 $$ \mathrm{cyt}{c}_1-{\mathrm{Fe}}^{2+}+\mathrm{cyt}{c}_2-{\mathrm{Fe}}^{3+}\to \mathrm{cyt}{c}_1-{\mathrm{Fe}}^{3+}+\mathrm{cyt}{c}_2-{\mathrm{Fe}}^{2+} $$

4 $$ \mathrm{cyt}{c}_2-{\mathrm{Fe}}^{2+}\to \mathrm{cyt}{c}_2-{\mathrm{Fe}}^{3+}+{\mathrm{e}}^{-} $$

In the last step, equation (4), cyt *c*_2_-Fe^2+^ is re-oxidised to cyt *c*_2_-Fe^3+^ at the electrode surface releasing the 1^st^ e^-^. The FADH^.^ radical formed in equation (2) is reoxidised to FAD by cyt *c*_1_-Fe^3+^ in equation (5) and the last two steps shown above (equations (3-4)) are repeated a second time for the regeneration of FAD and the protein in its native state, as follows (equations 5-7):

5 $$ {\mathrm{FADH}}^{\cdotp }+\mathrm{cyt}{c}_1-{\mathrm{Fe}}^{3+}\to \mathrm{FAD}+\mathrm{cyt}{c}_1-{\mathrm{Fe}}^{2+} $$

6 $$ \mathrm{cyt}{c}_1-{\mathrm{Fe}}^{2+}+\mathrm{cyt}{c}_2-{\mathrm{Fe}}^{3+}\to \mathrm{cyt}{c}_1-{\mathrm{Fe}}^{3+}+\mathrm{cyt}{c}_2-{\mathrm{Fe}}^{2+} $$

7 $$ \mathrm{cyt}{c}_2-{\mathrm{Fe}}^{2+}\to \mathrm{cyt}{c}_2-{\mathrm{Fe}}^{3+}+{\mathrm{e}}^{-} $$

where equations (6) and (7) are equal to equations (3) and (4), respectively. Equation (7) yields the 2^nd^ e- to the electrode.

The oxidation current for d-(-)-fructose at a FDH-modified electrode can be limited by the mass transfer of d-(-)-fructose to the electrode and/or by the kinetics of the enzymatic reaction. The measured current, *I*, is a combination of both the mass transfer–limited current, *I*_lim_, the kinetically limited current, *I*_kin_, and the current related to the interfacial electron transfer, *I*_E_, according to Eq. (8):

8 $$ \frac{1}{I}=\frac{1}{I_{\mathrm{lim}}}+\frac{1}{I_{\mathrm{kin}}}+\frac{1}{I_{\mathrm{E}}} $$

The mass transfer–limited current consists of the current observed when the d-(-)-fructose is consumed by the enzyme reaction much faster than d-(-)-fructose which is transported to the electrode surface. For a rotating disk electrode (RDE), the mass transfer–limited current depends on the angular velocity (*ω*) and the bulk concentration of d-(-)-fructose (c*) according to the Levich equation [70], as follows in Eq. (9a):

9a $$ {I_{\mathrm{lim}}}^{\mathrm{planar}}=0.620 nFc\ast {D}^{2/3}{A}_{\mathrm{geo}}{v}^{-1/6}\ {\omega}^{1/2} $$

where *n* and *F* have their usual meanings, *D* is the diffusion coefficient for d-(-)-fructose (7 × 10^−6^ cm^2^ s^−1^ [71]), *A* is the geometrical area of the electrode (0.073 cm^2^), and *v* is the kinematic viscosity of water (0.01 cm^2^ s^−1^).

Moreover, the mass transfer–limited current was evaluated also by using flow-through amperometry in a wall jet cell, for which the equation derived by Yamada and Matsuda can be applied (Eq. (9b)) [72]:

9b $$ {I_{\mathrm{lim}}}^{\mathrm{planar}}=0.898 nFc\ast {D}^{2/3}{A_{\mathrm{geo}}}^{3/8}{v}^{-5/12}{V}^{3/4}{a}^{-1/2} $$

where *V* is the volumetric flow rate and *a* is the radius of the capillary nozzle.

In this regard, we assumed that the AuNTr and the AuNSphs have a different self-packing pattern onto the electrode surface resulting in a different real surface area [73]. This can be determined by scanning the electrodes in H_2_SO_4_ and integrating the area under the wave for formation of gold oxide (data not shown). The real surface area (*A*_real_) resulted to be 4.6 ± 0.3 cm^2^ and 1.1 ± 0.2 cm^2^ for the AuNTr and the AuNSph modified electrodes, respectively. After this theoretical consideration, both Eqs. (9a) and (9b) were re-formulated as follows:

10a $$ \frac{I_{\mathrm{lim}}^{\mathrm{real}}}{I_{\mathrm{lim}}^{\mathrm{planar}}}=\frac{0.620 nF{c}^{\ast }{D}^{2/3}{A}_{\mathrm{real}}{v}^{-1/6}{\omega}^{1/2}}{0.620 nF{c}^{\ast }{D}^{2/3}{A}_{\mathrm{geo}}{v}^{-1/6}{\omega}^{1/2}} $$

10b $$ \frac{I_{\mathrm{lim}}^{\mathrm{real}}}{I_{\mathrm{lim}}^{\mathrm{planar}}}=\frac{0.898 nF{c}^{\ast }{D}^{2/3}{A}_{\mathrm{real}}^{3/8}{v}^{-5/12}{V}^{3/4}{a}^{-1/2}}{0.898 nF{c}^{\ast }{D}^{2/3}{A}_{\mathrm{geo}}^{3/8}{v}^{-5/12}{V}^{3/4}{a}^{-1/2}} $$

where *n*, *F*, c*, *D*, *v*, *V*, *a*, and *ω* have their usual meanings while *A*_real_/*A*_geo_ is the roughness factor calculated for the two different modified electrodes.

**Fig. 3:**
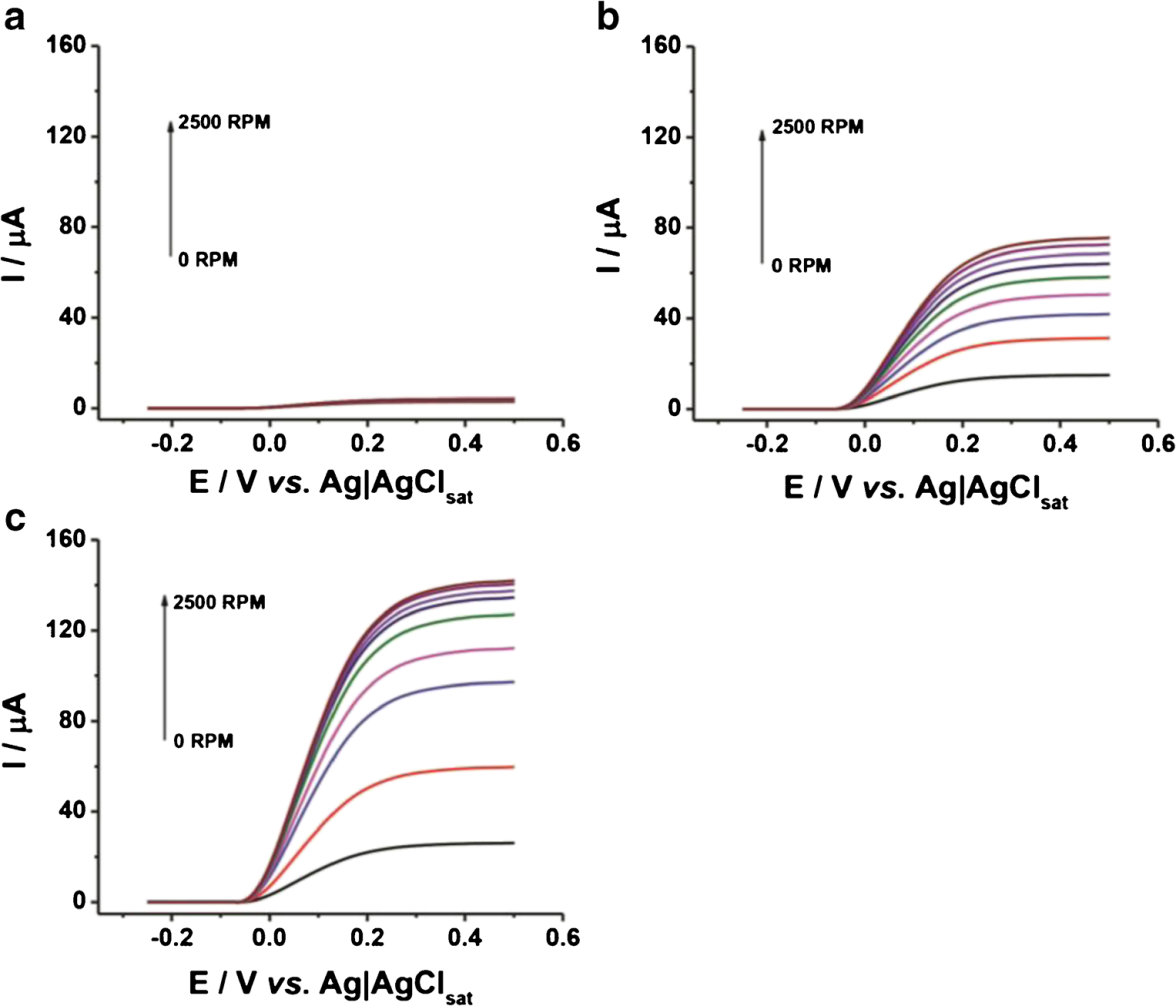
LSVs performed in 50 mM NaAc buffer pH 4.5 in presence of 1 mM d-(-)-fructose at different rotation speeds from 0 rpm to 2500 rpm. **a** FDH/G, **b** FDH/AuNSphs/G, and **c** FDH/AuNTrs/G at scan rate 5 mV s^−1^. The measurements were carried out after 20 min N_2_ degassing and *T* = 25 °C

**Fig. 4:**
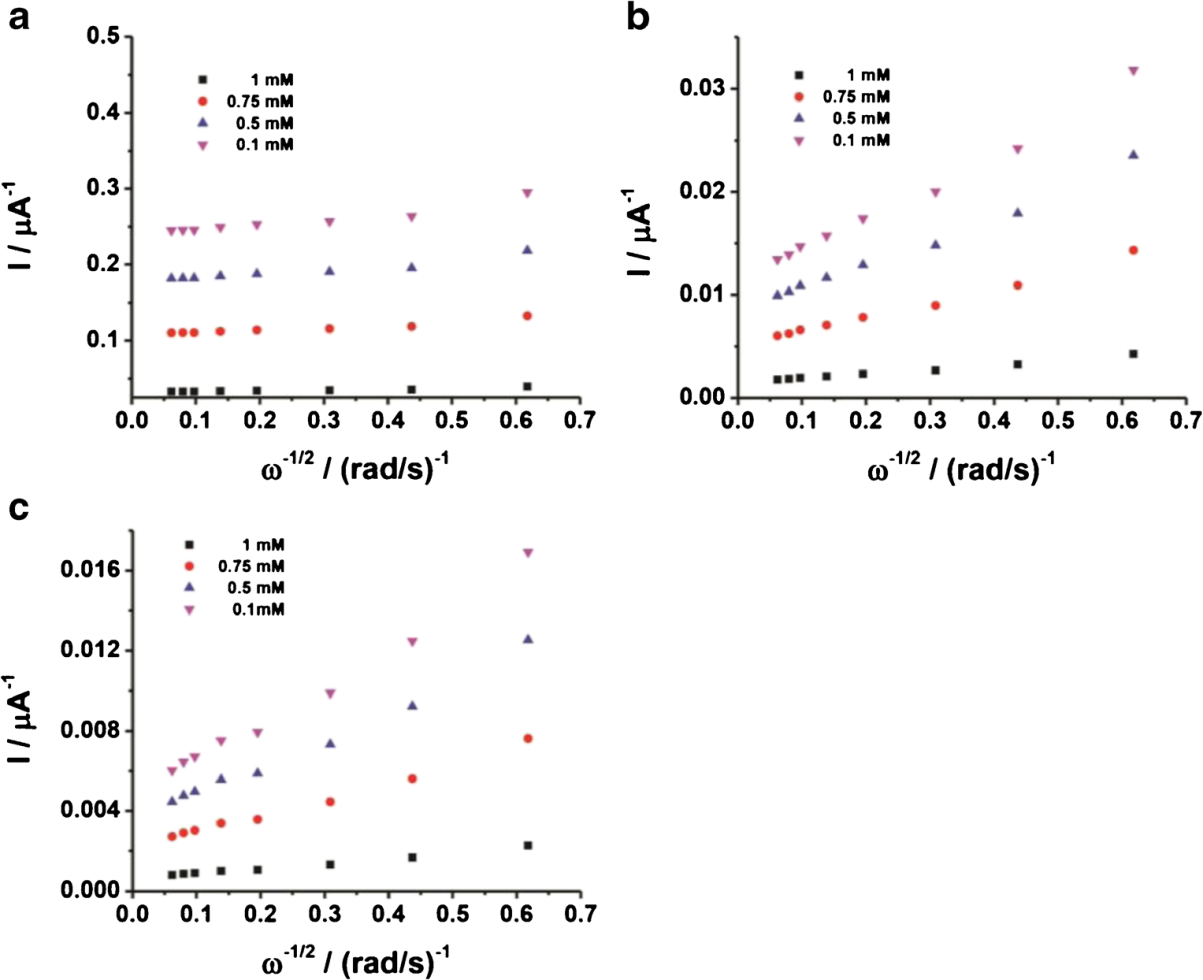
Koutecky-Levich plots in 50 mM NaAc buffer pH 4.5 in presence of 0.1 mM (purple), 0.5 mM (blue), 0.75 mM (red), and 1 mM d-(-)-fructose at different rotation speed from 0 rpm to 2500 rpm for **a** FDH/G, **b** FDH/AuNSphs/G, and **c** FDH/AuNTrs/G at scan rate 5 mV s^−1^. The measurements were carried out after 20 min N_2_ degassing and *T* = 25 °C. The current values were detected at *E* = + 0.4 V vs. Ag|AgCl_sat_

Rotating linear sweep voltammograms (RLSVs) for all the modified electrodes (viz. FDH/G, FDH/AuNSphs/G, and FDH/AuNTrs/G), obtained in presence of 1 mM d-(-)-fructose, are reported in Fig. 3a–c. As can be seen in Fig. 3, d-(-)-fructose oxidation at all modified electrodes resulted in a mass transfer–limited reaction (highly dependent from rotation speed). For a more deeper evaluation of the limiting steps of the performance of the RDE, the currents measured at different rotation speeds were as usually plotted in Koutecky-Levich coordinates (1/1 vs. *ω*^−1/2^) [74]. The d-(-)-fructose oxidation currents obtained with one FDH/G, FDH/AuNTrs/G, and FDH/AuNSphs/G at different [d-(-)-fructose] and *ω* are presented as Koutecky-Levich plots in Fig. 4a–c, for FDH/G, FDH/AuNTrs/G, and FDH/AuNSphs/G, respectively. Nevertheless, we applied the following Koutecky-Levich equation (Eq. (11)):

11 $$ \frac{1}{I}=\frac{1}{0.620 nF{c}^{\ast }{D}^{2/3}\left({A}_{\mathrm{geo}}\right){v}^{-1/6}{\omega}^{1/2}}+\frac{1}{nF\left(\raisebox{1ex}{${A}_{\mathrm{real}}$}\!\left/ \!\raisebox{-1ex}{${A}_{\mathrm{geo}}$}\right.\right)\Gamma {k}_{\mathrm{cat}}{c}^{\ast }}+\frac{1}{nF\left(\raisebox{1ex}{${A}_{\mathrm{real}}$}\!\left/ \!\raisebox{-1ex}{${A}_{\mathrm{geo}}$}\right.\right)\Gamma \left({k}_{s1}+{k}_{s2}\right)} $$

**Fig. 5:**
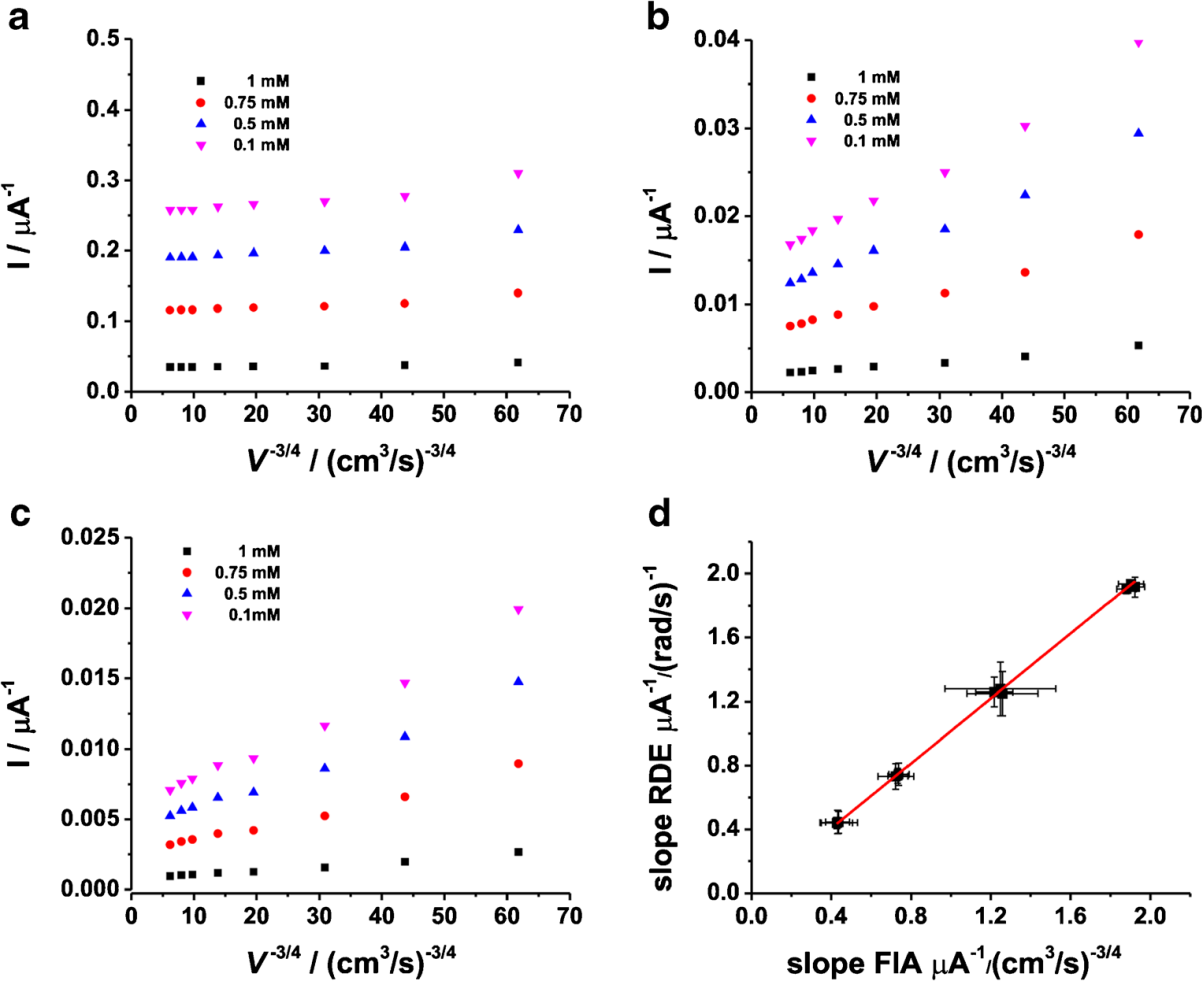
Corresponding plots for the wall jet system performed in 50 mM NaAc buffer pH 4.5 in presence of 0.1 mM (purple), 0.5 mM (blue), 0.75 mM (red), and 1 mM d-(-)-fructose at different flow rates from 0.05 to 2 mL/min for **a** FDH/G, **b** FDH/AuNSphs/G, and **c** FDH/AuNTrs/G. The measurements were carried out by applying *E* = + 0.4 V vs. Ag|AgCl_sat_. **d** Correlation plot between the slopes determined for RDE and FIA data obtained for FDH/AuNTrs/G

It can be seen that the electrode current depends on the *ω* (which is the criterion for diffusion limitation in the bioelectrocatalytic oxidation of d-(-)-fructose) in the range 50–400 μM. However, the data obtained for the FDH/G were fitted according to Eq. (10a) considering the graph 1/1_lim_ vs. [d-(-)-fructose] (data not shown), while the data for FDH/AuNTrs/G and FDH/AuNSphs/G well fitted (Eq. (10a)) taking into account the same graph. In this graph, it should be considered that the slope is proportional to the number of electrons transferred per molecule of d-(-)-fructose oxidized at the modified electrode, which was found to be 1.86 ± 0.02 for FDH/G, 1.93 ± 0.14 for FDH/AuNSphs/G, and 1.89 ± 0.20 for FDH/AuNTrs/G, values actually close to the theoretical value of 2, while *k*_cat_ (s^−1^) can be calculated from the intercept. The mass transfer–limited currents were also evaluated by considering Eq. (10b) valid for flow-through setup obtaining similar results. The equivalent Koutecky-Levich plots obtained by the flow-through setup for FDH/G, FDH/AuNSphs/G, and FDH/AuNTrs/G, respectively, are reported in Figs. 5a–c. These results were in great agreement with those reported for RDE as confirmed from the correlation factor *R*^2^ = 0.98, as shown in the correlation graph reported in Fig. 5d.

### The dependence of kinetically limited current on the nanoparticle shape: rotating disk electrode and flow through amperometry studies

Before discussing the data on the kinetically limited current, we should consider the equation for the kinetically limited current, as follows (Eq. (12)):

12 $$ \frac{1}{I_{\mathrm{kin}}}+\frac{1}{I_{\mathrm{E}}}=\frac{1}{nFA\Gamma}\frac{1}{\left({k}_{\mathrm{cat}}{c}^{\ast }+{k}_{s1}+{k}_{s2}\right)} $$

The equation above, Eq. (12), is valid for the FDH/G electrode, while for FDH/AuNTrs/G and FDH/AuNSphs/G, a contribution on the enhancement of the electrode area should be considered; therefore, Eq. (12) can be rearranged as follows (Eq. (13)):

13 $$ \frac{1}{I_{\mathrm{kin}}}+\frac{1}{I_{\mathrm{E}}}=\frac{1}{nF\left(\raisebox{1ex}{${A}_{\mathrm{real}}$}\!\left/ \!\raisebox{-1ex}{${A}_{\mathrm{geo}}$}\right.\right)\Gamma}\frac{1}{\left({k}_{\mathrm{cat}}{c}^{\ast }+{k}_{s1}+{k}_{s2}\right)} $$

At this stage, we need to further approximate the system in order to determine the catalytic constant, *k*_cat_, and the heterogeneous electron transfer rate constant, *k*_St_, considering that the internal electron transfer is not the rate-limiting step in the overall electron transfer mechanism [75]. Thus, we would consider reactions (1), (5), and (9) in order to deeply evaluate the effect of shape of the nanoparticles on *k*_cat_ and *k*_St_. Finally, Eq. (13) was rearranged as follows (Eq. (14)):

14 $$ \frac{1}{I_{\mathrm{kin}}}+\frac{1}{I_{\mathrm{E}}}=\frac{1}{nF\left(\raisebox{1ex}{${A}_{\mathrm{real}}$}\!\left/ \!\raisebox{-1ex}{${A}_{\mathrm{geo}}$}\right.\right)\Gamma}\frac{1}{\left({k}_{\mathrm{cat}}{c}^{\ast }+{k}_{\mathrm{St}}\right)} $$

At limiting step, by considering 1/*I*_E_ = 0. The experimental conditions at which 1/*I*_E_ = 0 are low substrate concentration (*C*), rotation speed (*ω*) of the electrode, and applying sufficiently large an electrochemical driving force |*E*-*E*^0^′|. In this way, it was possible to increase the influence of the Levich and the enzymatic component in Eqs. (8) and (14); therefore, the kinetics contribution (1/*I*_E_) would be negligible (1/*I*_E_ = 0). Therefore, we can simplify Eq. (14) as follows (Eq. (15)):

15 $$ \frac{1}{I_{\mathrm{kin}}}=\frac{1}{nF\left(\raisebox{1ex}{${A}_{\mathrm{real}}$}\!\left/ \!\raisebox{-1ex}{${A}_{\mathrm{geo}}$}\right.\right)\Gamma}\frac{1}{k_{\mathrm{cat}}{c}^{\ast }} $$

Kinetically limited currents of the oxidation of d-(-)-fructose can be evaluated from the intercepts of the Koutecky-Levich plots. According to the mathematical expression for *I*_kin_ (Eq. (14)), the slope of this plot is proportional to the rate of the reaction between d-(-)-fructose and FDH (constant *k*_cat_ in reaction (1)), while the intercept is proportional to the heterogeneous electron transfer (constant *k*_St_ in reactions (2–4) and (5–7)) between reduced FDH and the graphite modified surface (AuNTrs and AuNSphs). To evaluate the rates of these reactions, the surface concentration of FDH on the graphite modified electrode must be known. The theoretical surface coverage resulted in 0.80 nmol cm^−2^ was considered in this paper for all the modified electrodes, namely FDH/G, FDH/AuNSphs/G, and FDH/AuNTrs/G. Therefore, it was possible to estimate *k*_1_, the kinetic constant for reaction (1), and the heterogeneous electron transfer (constant *k*_St_ in reactions (2–4) and (5–7)). The results calculated for FDH/G, FDH/AuNSphs/G, and FDH/AuNTrs/G are summarized in Table 1. From these results, it is possible to see that the shape of the NPs had no effect on the catalytic constant (*k*_cat_), while the *k*_St_ for FDH/AuNTrs/G calculated as 3.8 ± 0.3 s^−1^ resulted in a 5 times higher value compared with both the NPless graphite electrode 0.7 ± 0.1 s^−1^ and the AuNSphs/G modified electrode 0.9 ± 0.1 s^−1^. These results are probably related to the shape of the NPs because the AuNTrs due to their triangular geometry have different self-packing mechanism compared with the spherical ones ensuring a higher real surface area. Nevertheless, it should be taken into account also the interaction between the enzyme molecules and the NPs highlighting that the interaction enzyme-NPs can occur on the edge of the triangle while the spherical shape is limiting the number of enzyme molecules interacting with each NP.

**Table 1:** Kinetic parameters calculated from the RDE data for FDH/G, FDH/AuNSphs/G, and FDH/AuNTrs/G. *n* is equal to the number of electrons participating in the reaction

	*n*	*k*_cat_ (s^−1^)	*k*_St_ (s^−1^)
FDH/G	1.86 ± 0.02	2.6 ± 0.1	0.7 ± 0.1
FDH/AuNSphs/G	1.93 ± 0.14	2.8 ± 0.1	0.9 ± 0.1
FDH/AuNTrs/G	1.89 ± 0.20	2.9 ± 0.3	3.8 ± 0.3

As further investigations, we studied also the storage and operational stability of the proposed modified electrodes, namely FDH/G, FDH/AuNSphs/G, and FDH/AuNTrs/G, and the results are reported in Fig. S2A and B (see ESM), showing quite a stable signal for 24 h of continuous injections of substrate into the flow system, while in the storage stability test, it was possible to observe a significant drop in the retained current values of approximately 65% for FDH/AuNTrs/G, 72% for FDH/AuNSphs/G, and 70% for FDH/G (compared with the initial current value) achieved after 20 days.

## Conclusions

Finally, we have unequivocally demonstrated that the shape of the NPs had a crucial effect on the catalytic current related to the oxidation of d-(-)-fructose to 5-keto-d-(-)-fructose occurring at the FDH-modified electrode surface. In particular, we have shown that AuNTrs have a higher effect compared with the spherical one. The effect was deeply investigated for each contribution to the total catalytic current (*I*), namely mass transfer–limited current (*I*_lim_), and kinetically limited current (*I*_kin_), by using two different approaches: RDE and flow through amperometry. The shape of the NPs had no effect on the catalytic constant (*k*_cat_), while the *k*_St_ for FDH/AuNTrs/G resulted in a 5 times higher value compared with both the NPless graphite electrode and the AuNSphs/G modified electrode. These results can probably be ascribed to the shape because with the triangular NPs, the interaction enzyme-NPs can occur on the edge of the triangle, whereas for the spherical shape, the number of enzyme molecules interacting with NPs is limited. These findings would be of fundamental interest to study the kinetic mechanism of FDH and to develop highly efficient 3rd-generation biosensors and EFC bioanode based on metal NPs of various sizes [17] and shapes.

## Electronic supplementary material

ESM 1 (PDF 197 kb)

## References

[1] Zhu CZ, Yang GH, Li H, Du D, Lin YH (2015). Electrochemical sensors and biosensors based on nanomaterials and nanostructures. Anal Chem.

[2] Arduini F, Micheli L, Moscone D, Palleschi G, Piermarini S, Ricci F, Volpe G (2016). Electrochemical biosensors based on nanomodified screen-printed electrodes: recent applications in clinical analysis. Trends Anal Chem.

[3] Bollella P, Gorton L, Antiochia R (2018). Direct electron transfer of dehydrogenases for development of 3rd generation biosensors and enzymatic fuel cells. Sensors.

[4] Bollella P, Ludwig R, Gorton L (2017). Cellobiose dehydrogenase: insights on the nanostructuration of electrodes for improved development of biosensors and biofuel cells. Appl Mater Today.

[5] Bollella P, Fusco G, Stevar D, Gorton L, Ludwig R, Ma S, Boer H, Koivula A, Tortolini C, Favero G (2018). A glucose/oxygen enzymatic fuel cell based on gold nanoparticles modified graphene screen-printed electrode. Proof-of-concept in human saliva. Sens Actuat B.

[6] Ghindilis AL, Atanasov P, Wilkins E (1997). Enzyme-catalyzed direct electron transfer: fundamentals and analytical applications. Electroanalysis.

[7] Ferapontova EE, Shleev S, Ruzgas T, Stoica L, Christenson A, Tkac J, Yaropolov AI, Gorton L (2005). Direct electrochemistry of proteins and enzymes. Perspect Bioanal.

[8] Bollella P, Mazzei F, Favero G, Fusco G, Ludwig R, Gorton L, Antiochia R (2017). Improved DET communication between cellobiose dehydrogenase and a gold electrode modified with a rigid self-assembled monolayer and green metal nanoparticles: the role of an ordered nanostructuration. Biosens Bioelectron.

[9] Zafar MN, Aslam I, Ludwig R, Xu G, Gorton L (2019). An efficient and versatile membraneless bioanode for biofuel cells based on *Corynascus thermophilus* cellobiose dehydrogenase. Electrochim Acta.

[10] Kizling M, Draminska S, Stolarczyk K, Tammela P, Wang Z, Nyholm L, Bilewicz R (2015). Biosupercapacitors for powering oxygen sensing devices. Bioelectrochemistry.

[11] Kizling M, Dzwonek M, Więckowska A, Bilewicz R (2018). Size does matter—mediation of electron transfer by gold clusters in bioelectrocatalysis. Chem Cat Chem.

[12] Kizling M, Rekorajska A, Krysinski P, Bilewicz R (2018). Magnetic-field-induced orientation of fructose dehydrogenase on iron oxide nanoparticles for enhanced direct electron transfer. Electrochem Commun.

[13] Shumakovich G, Otrokhov G, Vasil’eva I, Pankratov D, Morozova O, Yaropolov A (2012). Laccase-mediated polymerization of 3, 4-ethylenedioxythiophene (EDOT). J Mol Catal B.

[14] Pankratov DV, Zeifman YS, Morozova OV, Shumakovich GP, Vasil’eva IS, Shleev S, Popov VO, Yaropolov AI (2013). A comparative study of biocathodes based on multiwall carbon nanotube buckypapers modified with three different multicopper oxidases. Electroanalysis.

[15] Frasca S, Rojas O, Salewski J, Neumann B, Stiba K, Weidinger IM, Tiersch B, Leimkuehler S, Koetz J, Wollenberger U (2012). Human sulfite oxidase electrochemistry on gold nanoparticles modified electrode. Bioelectrochemistry.

[16] Pankratov D, Sundberg R, Suyatin DB, Sotres J, Barrantes A, Ruzgas T, Maximov I, Montelius L, Shleev S (2014). The influence of nanoparticles on enzymatic bioelectrocatalysis. RSC Adv.

[17] Kizling M, Dzwonek M, Wieckowska A, Bilewicz R. Gold nanoparticles in bioelectrocatalysis–the role of nanoparticle size. Curr Opin Electrochem. 2018.

[18] Kamitaka Y, Tsujimura S, Kano K (2006). High current density bioelectrolysis of D-fructose at fructose dehydrogenase-adsorbed and Ketjen black-modified electrodes without a mediator. Chem Lett.

[19] Kamitaka Y, Tsujimura S, Setoyama N, Kajino T, Kano K (2007). Fructose/dioxygen biofuel cell based on direct electron transfer-type bioelectrocatalysis. Phys Chem Chem Phys.

[20] Kawai S, Goda-Tsutsumi M, Yakushi T, Kano K, Matsushita K (2013). Heterologous overexpression and characterization of a flavoprotein-cytochrome *c* complex fructose dehydrogenase of *Gluconobacter japonicus* NBRC3260. Appl Environ Microbiol.

[21] Kawai S, Yakushi T, Matsushita K, Kitazumi Y, Shirai O, Kano K (2014). The electron transfer pathway in direct electrochemical communication of fructose dehydrogenase with electrodes. Electrochem Commun.

[22] Hamano Y, Tsujimura S, Shirai O, Kano K (2012). Micro-cubic monolithic carbon cryogel electrode for direct electron transfer reaction of fructose dehydrogenase. Bioelectrochemistry.

[23] Bollella P, Hibino Y, Kano K, Gorton L, Antiochia R (2018). Highly sensitive membraneless fructose biosensor based on fructose dehydrogenase immobilized onto aryl thiol modified highly porous gold electrode: characterization and application in food samples. Anal Chem.

[24] Kizling M, Bilewicz R (2018). Fructose dehydrogenase electron transfer pathway in bioelectrocatalytic reactions. Chem Electro Chem.

[25] Hibino Y, Kawai S, Kitazumi Y, Shirai O, Kano K (2017). Construction of a protein-engineered variant of D-fructose dehydrogenase for direct electron transfer-type bioelectrocatalysis. Electrochem Commun.

[26] Bollella P, Hibino Y, Kano K, Gorton L, Antiochia R (2018). Enhanced direct electron transfer of fructose dehydrogenase rationally immobilized on a 2-aminoanthracene diazonium cation grafted single-walled carbon nanotube based electrode. ACS Catal.

[27] Kinnear KT, Monbouquette HG (1997). An amperometric fructose biosensor based on fructose dehydrogenase immobilized in a membrane mimetic layer on gold. Anal Chem.

[28] Tominaga M, Nomura S, Taniguchi I (2009). D-Fructose detection based on the direct heterogeneous electron transfer reaction of fructose dehydrogenase adsorbed onto multi-walled carbon nanotubes synthesized on platinum electrode. Biosens Bioelectron.

[29] Xia H, Hibino Y, Kitazumi Y, Shirai O, Kano K (2016). Interaction between D-fructose dehydrogenase and methoxy-substituent-functionalized carbon surface to increase productive orientations. Electrochim Acta.

[30] Sakinyte I, Barkauskas J, Gaidukevic J, Razumiene J (2015). Thermally reduced graphene oxide: the study and use for reagentless amperometric D-fructose biosensors. Talanta.

[31] Tsujimura S, Nishina A, Kamitaka Y, Kano K (2009). Coulometric D-fructose biosensor based on direct electron transfer using D-fructose dehydrogenase. Anal Chem.

[32] Parellada J, Domínguez E, Fernandez VM (1996). Amperometric flow injection determination of fructose in honey with a carbon paste sensor based on fructose dehydrogenase. Anal Chim Acta.

[33] Murata K, Suzuki M, Kajiya K, Nakamura N, Ohno H (2009). High performance bioanode based on direct electron transfer of fructose dehydrogenase at gold nanoparticle-modified electrodes. Electrochem Commun.

[34] Darder M, Casero E, Pariente F, Lorenzo E (2000). Biosensors based on membrane-bound enzymes immobilized in a 5-(octyldithio)-2-nitrobenzoic acid layer on gold electrodes. Anal Chem.

[35] Siepenkoetter T, Salaj-Kosla U, Magner E (2017). The immobilization of fructose dehydrogenase on nanoporous gold electrodes for the detection of fructose. ChemElectroChem.

[36] Antiochia R, Gorton L (2014). A new osmium-polymer modified screen-printed graphene electrode for fructose detection. Sens. Actuat. B.

[37] Antiochia R, Vinci G, Gorton L (2013). Rapid and direct determination of fructose in food: a new osmium-polymer mediated biosensor. Food Chem.

[38] Nicholas P, Pittson R, Hart JP (2018). Development of a simple, low cost chronoamperometric assay for fructose based on a commercial graphite-nanoparticle modified screen-printed carbon electrode. Food Chem.

[39] Biscay J, Costa Rama E, Gonzalez Garcia MB, Reviejo AJ, Pingarron Carrazon JM, Garcia AC (2012). Amperometric fructose sensor based on ferrocyanide modified screen-printed carbon electrode. Talanta.

[40] Damar K, Demirkol DO (2011). Modified gold surfaces by poly(amidoamine) dendrimers and fructose dehydrogenase for mediated fructose sensing. Talanta.

[41] Trivedi UB, Lakshminarayana D, Kothari IL, Patel PB, Panchal CJ (2009). Amperometric fructose biosensor based on fructose dehydrogenase enzyme. Sens. Actuat. B.

[42] Montanez-Soto JL, Alegret S, Salazar-Montoya JA, Ramos-Ramirez EG (2006). A new amperometric biosensor for fructose determination based on epoxy-graphite-TTF-TCNQ-FDH-biocomposite. Eur Food Res Technol.

[43] Antiochia R, Lavagnini I, Magno F (2004). Amperometric mediated carbon nanotube paste biosensor for fructose determination. Anal Lett.

[44] Tkac J, Vostiar I, Gemeiner P, Sturdik E (2002). Stabilization of ferrocene leakage by physical retention in a cellulose acetate membrane. The fructose biosensor. Bioelectrochemistry.

[45] Watanabe S, Kubo I (2002). Fructose biosensor based on D-fructose dehydrogenase and phenanthroline cobalt complex as a mediator. Electrochemistry.

[46] Garcia CAB, Neto GD, Kubota LT (1998). New fructose biosensors utilizing a polypyrrole film and D-fructose 5-dehydrogenase immobilized by different processes. Anal Chim Acta.

[47] Garcia CAB, Neto GD, Kubota LT, Grandin LA (1996). A new amperometric biosensor for fructose using a carbon paste electrode modified with silica gel coated with Meldola’s blue and fructose 5-dehydrogenase. J Electroanal Chem.

[48] Paredes PA, Parellada J, Fernandez VM, Katakis I, Domínguez E (1997). Amperometric mediated carbon paste biosensor based on D-fructose dehydrogenase for the determination of fructose in food analysis. Biosens Bioelectron.

[49] Pingarrón JM, Yáñez-Sedeño P, González-Cortés A (2008). Gold nanoparticle-based electrochemical biosensors. Electrochim Acta.

[50] Yanez-Sedeno P, Pingarron J (2005). Gold nanoparticle-based electrochemical biosensors. Anal Bioanal Chem.

[51] Guo S, Wang E (2007). Synthesis and electrochemical applications of gold nanoparticles. Anal Chim Acta.

[52] Kimling J, Maier M, Okenve B, Kotaidis V, Ballot H, Plech A (2006). Turkevich method for gold nanoparticle synthesis revisited. J Phys Chem B.

[53] Hussain I, Graham S, Wang Z, Tan B, Sherrington DC, Rannard SP, Cooper AI, Brust M (2005). Size-controlled synthesis of near-monodisperse gold nanoparticles in the 1−4 nm range using polymeric stabilizers. J Am Chem Soc.

[54] Johnson CJ, Dujardin E, Davis SA, Murphy CJ, Mann S (2002). Growth and form of gold nanorods prepared by seed-mediated, surfactant-directed synthesis. J Mater Chem.

[55] Engelbrekt C, Sørensen KH, Zhang J, Welinder AC, Jensen PS, Ulstrup J (2009). Green synthesis of gold nanoparticles with starch–glucose and application in bioelectrochemistry. J Mater Chem.

[56] Begum NA, Mondal S, Basu S, Laskar RA, Mandal D (2009). Biogenic synthesis of Au and Ag nanoparticles using aqueous solutions of black tea leaf extracts. Coll Surface B.

[57] Bollella P, Schulz C, Favero G, Mazzei F, Ludwig R, Gorton L, Antiochia R (2017). Green synthesis and characterization of gold and silver nanoparticles and their application for development of a third generation lactose biosensor. Electroanalysis.

[58] Ibano D, Yokota Y, Tominaga T (2003). Preparation of gold nanoplates protected by an anionic phospholipid. Chem Lett.

[59] Gao J, Bender CM, Murphy CJ (2003). Dependence of the gold nanorod aspect ratio on the nature of the directing surfactant in aqueous solution. Langmuir.

[60] Daniel M-C, Astruc D (2004). Gold nanoparticles: assembly, supramolecular chemistry, quantum-size-related properties, and applications toward biology, catalysis, and nanotechnology. Chem Rev.

[61] Park K, Koerner H, Vaia RA (2010). Depletion-induced shape and size selection of gold nanoparticles. Nano Lett.

[62] Appelqvist R, Marko-Varga G, Gorton L, Torstensson A, Johansson G (1985). Enzymatic determination of glucose in a flow system by catalytic oxidation of the nicotinamide coenzyme at a modified electrode. Anal Chim Acta.

[63] Liebig F, Sarhan RM, Prietzel C, Reinecke A, Koetz J (2016). “Green” gold nanotriangles: synthesis, purification by polyelectrolyte/micelle depletion flocculation and performance in surface-enhanced Raman scattering. RSC Adv.

[64] Scarabelli L, Coronado-Puchau M, Giner-Casares JJ, Langer J, Liz-Marzán LM (2014). Monodisperse gold nanotriangles: size control, large-scale self-assembly, and performance in surface-enhanced Raman scattering. ACS Nano.

[65] Hernández J, Solla-Gullón J, Herrero E, Aldaz A, Feliu JM (2007). Electrochemistry of shape-controlled catalysts: oxygen reduction reaction on cubic gold nanoparticles. J Phys Chem C.

[66] Streeter I, Compton RG (2007). Diffusion-limited currents to nanoparticles of various shapes supported on an electrode; spheres, hemispheres, and distorted spheres and hemispheres. J Phys Chem C.

[67] Ruzgas T, Gorton L, Emnéus J, Marko-Varga G (1995). Kinetic models of horseradish peroxidase action on a graphite electrode. J Electroanal Chem.

[68] Lindgren A, Munteanu F-D, Gazaryan IG, Ruzgas T, Gorton L (1998). Comparison of rotating disk and wall-jet electrode systems for studying the kinetics of direct and mediated electron transfer for horseradish peroxidase on a graphite electrode. J Electroanal Chem.

[69] Karyakin AA, Karyakina EE, Gorton L (1998). The electrocatalytic activity of Prussian blue in hydrogen peroxide reduction studied using a wall-jet electrode with continuous flow. J Electroanal Chem.

[70] Bard AJ (1980). LR Faulkner electrochemical methods.

[71] M. Rampp, C. Buttersack, H.-D. Lüdemann, c, T-dependence of the viscosity and the self-diffusion coefficients in some aqueous carbohydrate solutions, Carbohydr Res, 328 (2000) 561–572. 10.1016/s0008-6215(00)00141-5 11093712

[72] Yamada J, Matsuda H (1973). Limiting diffusion currents in hydrodynamic voltammetry: III. Wall jet electrodes. J Electroanal Chem.

[73] Masa J, Batchelor-McAuley C, Schuhmann W, Compton RG (2014). Koutecky-Levich analysis applied to nanoparticle modified rotating disk electrodes: electrocatalysis or misinterpretation. Nano Res.

[74] Scott DL, Bowden EF (1994). Enzyme-substrate kinetics of adsorbed cytochrome c peroxidase on pyrolytic graphite electrodes. Anal Chem.

[75] Coughlan MP, Rajagopalan K (1980). The kinetic mechanism of xanthine dehydrogenase and related enzymes. FEBS J.

