# Supplementary material for: The influence of the shape of Au nanoparticles on the catalytic current of fructose dehydrogenase
Source: Anal Bioanal Chem. 2019 Jul 8;411(29):7645–57. doi: 10.1007/s00216-019-01944-6 (PMC6881425; doi:10.1007/s00216-019-01944-6)
Supplement: Supplementary file 1 — (PDF 197 kb) [file 216_2019_1944_MOESM1_ESM.pdf]

Analytical and Bioanalytical Chemistry

Electronic Supplementary Material

The influence of the shape of Au nanoparticles on the catalytic current of fructose dehydrogenase

Paolo Bollella, Yuya Hibino, Paolo Conejo-Valverde, Jackeline Soto-Cruz, Julián Bergueiro, Marcelo Calderón, Oscar Rojas-Carrillo, Kenji Kano, Lo Gorton

Spectrophotometric kinetic assay procedure

the activity of the DHFDH domain was monitored measuring the time-dependent variation of the absorbance at λ = 520 nm (ε = 6.9 mM-1 cm-1) of a mixture containing 100 μL of 10 mM D-(–)-fructose, 780 μL of 50 mM NaAc buffer, 20 μL of enzyme solution, and 100 μL of 3 mM DCIP as two- electrons/two protons acceptor [1].

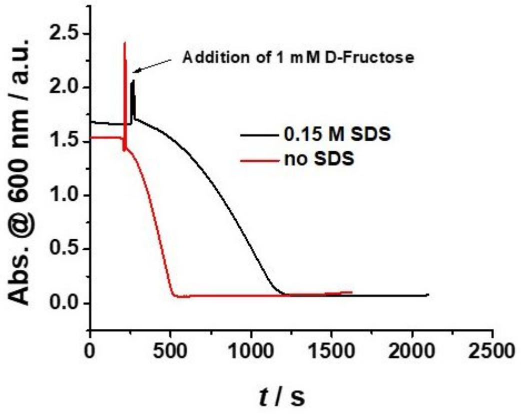

Fig. S1 Spectrophotometric kinetic measurements of FDH enzymatic activity using 2,6-dichloroindophenol (DCIP, 0.06 mM) as electron acceptor performed in 50 mM NaAc buffer at pH 4.5 (adding 1 mM D-(-)-fructose as substrate) in the absence of SDS (red line) and in the presence of 0.15 M SDS (black line)

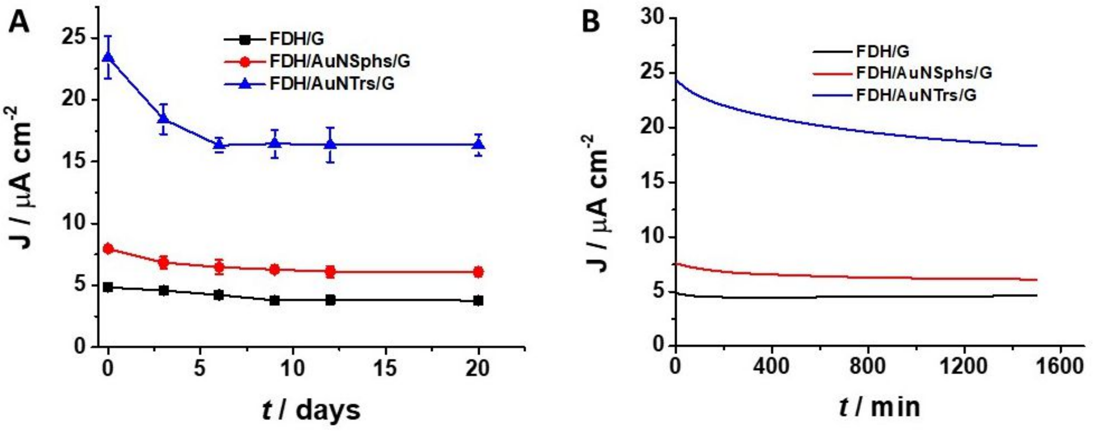

Fig. S2 (A) Storage stability measurements performed in 50 mM NaAc buffer at pH 4.5 in the presence of 1 mM D-(-)-fructose for (black line) FDH/G, (red line) FDH/AuNSphs/G and (blue line) FDH/AuNTrs/G . The measurements were carried out by applying E = +0.4 V vs. Ag|AgCl$_{sat}$ at a flowrate of 0.5 mL min^{-1}. **(A)** Operational stability measurements performed in 50 mM NaAc buffer at pH 4.5 in the presence of 1 mM D-(-)-fructose for (black line) FDH/G, (red line) FDH/AuNSphs/G and (blue line) FDH/AuNTrs/G. The measurements were carried out by applying E = +0.4 V vs. Ag|AgCl$_{sat}$ at a flow rate of 0.5 mL min^{-1}

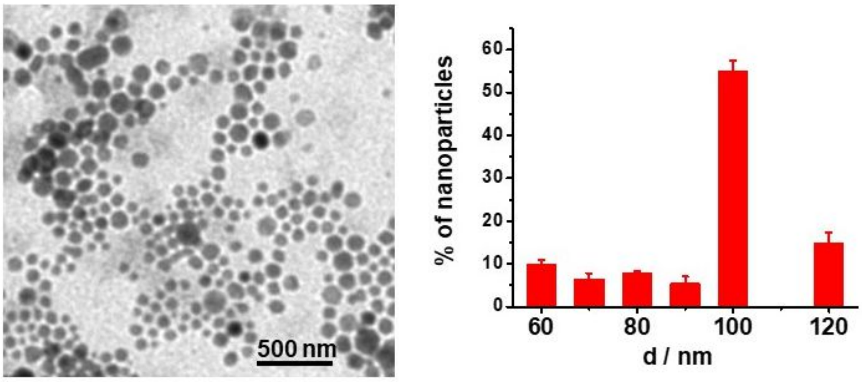

Fig. S3 TEM picture and size distribution of AuNSphs

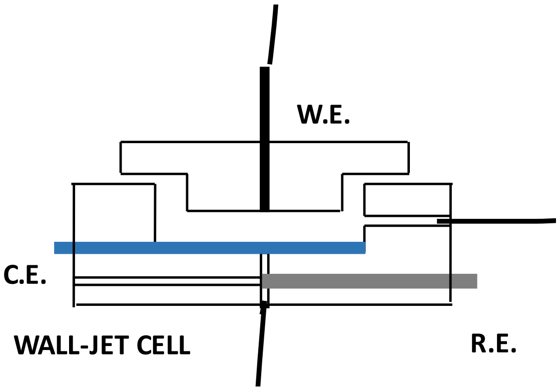

Fig. S4 Scheme of the wall-jet cell used for kinetic measurements

References

[1] Schulz C, Ludwig R, Micheelsen PO, Silow M, Toscano MD, Gorton L. Enhancement of enzymatic activity and catalytic current of cellobiose dehydrogenase by calcium ions. Electrochem Commun. 2012;17(Suppl. C):71–4.
